# Optimizing Adjuvant Therapy after Surgery for Colorectal Cancer Liver Metastases: A Systematic Review

**DOI:** 10.3390/jcm12062401

**Published:** 2023-03-20

**Authors:** Emmanouil Georgilis, Maria Gavriatopoulou, Diamantis I. Tsilimigras, Panagiotis Malandrakis, Theodosios Theodosopoulos, Ioannis Ntanasis-Stathopoulos

**Affiliations:** 1 Department of Clinical Therapeutics, School of Medicine, National and Kapodistrian University of Athens, 11528 Athens, Greece; 2 Department of Surgery, Division of Surgical Oncology, The Ohio State University Wexner Medical Center and James Comprehensive Cancer Center, Columbus, OH 43210, USA; 3 Second Department of Surgery, Aretaieion University Hospital, National and Kapodistrian University of Athens, 11528 Athens, Greece

**Keywords:** colorectal cancer liver metastases, liver metastasectomy, adjuvant therapy, systematic chemotherapy, hepatic arterial infusion chemotherapy, systematic review

## Abstract

The liver is the most common site of colorectal cancer metastatic spread. Although metastasectomy is the gold standard for fit patients with resectable colorectal cancer liver metastases (CRLMs), their management after surgical treatment remains controversial. The objective of this systematic review was to collate the currently available data of the agents used in the adjuvant setting in order to define the most optimal therapeutic strategy. A systematic review of the literature was conducted by searching PubMed/Medline and Cochrane library databases. We included studies that evaluated the efficacy, the tolerability and the safety profile of various chemotherapeutic agents that are used as adjuvant treatment after surgical resection of CRLMs. The outcomes of interest were regression-free survival (RFS), disease-free survival (DFS), overall survival (OS) and severe toxicities. From 543 initial articles, 29 publications with 7028 patients were finally included. In general, the results of the eligible studies indicated that adjuvant therapy after resection of CRLMs led to improved RFS/DFS rates, but this benefit did not contribute to a statistically significant prolongation of OS. Moreover, the choice of the therapeutic strategy, namely systematic or regional chemotherapy or the combination of both, did not seem to have a differential impact on patient outcomes. However, these results should be interpreted with caution since the majority of the chosen studies are of low or moderate quality. In this context, further high-quality clinical trials conducted on patient sub-populations with modern therapies are required in order to reduce in-study and between-study heterogeneity and determine which patients are expected to derive the maximum benefit from adjuvant therapy after surgery for CRLMs.

## 1. Introduction

Colorectal cancer (CRC) is the third most common cancer type and the second leading cause of cancer death worldwide. In 2020, 1,880,725 new cases and 915,880 new deaths were recorded [1]. About half of these cases would develop liver metastases, 25% of them synchronously, making the liver the most common site of metastatic spread [2,3]. This metastatic pattern could be attributed to the portal venous drainage of the colon to the liver.

Hepatic resection has become the standard management in patients with resectable colorectal liver metastases (CRLMs) [3,4], and it is associated with the best chance of long-term survival. Within the last few years, there has been a trend toward higher survival rates in patients with CRLMs due to more efficient chemotherapy regimens and new surgical techniques or strategies [4,5,6,7,8], leading to 5-year survival rates ranging from 46% to 58% and 10-year survival rates of greater than 20% in specialized centers [4,9,10,11]. Patients without recurrence beyond 10 years after resection of liver metastases could be considered potentially cured [12]. The modern and more sensitive imaging modalities, including hepatobiliary phase MRI and PET-CT scan, contributed to the aforementioned improvement in patient outcomes by enabling prompt metastatic detection in liver and leading to optimal decision-making about resection [13,14,15]. However, the recurrence rate remains high, even after curative R0 resection [16,17]. In an effort to decrease the recurrence of metastatic disease, many studies have investigated the efficacy of either systemic or regional chemotherapy or the combination of both methods following complete liver metastasectomy.

The typical systemic chemotherapy backbone comprises intravenous 5-FU, a fluoropyrimidine, used in various schemes and schedules with either oxaliplatin or irinotecan (FOLFOX or FOLFIRI regimens). These combinations provide a higher response rate and better progression-free and overall survival than fluoropyrimidine (5-FU/leucovorin) monotherapy [18,19]. The therapeutic armamentarium also includes oral chemotherapeutic regimens, such as the combination of the 5-FU prodrug tegafur, which is metabolized by the liver, and the competitive inhibitor of the main metabolizing enzyme of 5-fluorouracil, uracil (UTF regimen). In that way, the serum concentration of 5-fluorouracil is increased. UTF in combination with an oral folinic acid preparation (e.g., leucovorin) has been used as an adjuvant treatment for stage III CRC [20,21], and because of its oral and convenient administration, it may have practical advantages as suggested by previous trials [22,23]. Another oral agent is capecitabine, which can be used in place of 5-FU/leucovorin either alone [24] or combined with oxaliplatin (CAPOX regimen) [25], but less frequently with irinotecan because of early concerns that this scheme was more toxic than FOLFIRI [26]. 

An alternative option in the adjuvant setting also includes hepatic arterial infusion (HAI) chemotherapy. The biological rationale behind this therapeutic approach is that liver metastases receive blood solely from the hepatic artery, whereas normal hepatic parenchyma derives most of its supply from the portal vein [27]. During its process, intra-arterial regimens are administered continuously into the liver via a subcutaneous pump or an intra-arterial catheter. Its advantage in comparison with systemic chemotherapy is that it allows the administration of higher doses of chemotherapeutic agents, while the risk of systemic toxicities is minimized [28]. For instance, over 95% of floxuridine (FUDR), a metabolite of 5-FU and the most studied agent in this setting, is extracted by the liver during the first-pass metabolism, leading to a 400-fold increase in hepatic exposure [29]. In general, it is considered as a safe and feasible method in specialized centers [30]. 

Nevertheless, despite all the aforementioned therapeutic choices, the management of CRC patients after liver metastasectomy is still controversial and there is no standard treatment. Thus, the aim of this systematic review was to compare the efficacy, the tolerability and the safety profile of the available therapeutic regimens in order to provide a critical overview and determine the optimal clinical approach to adjuvant treatment after CRLM resection.

## 2. Materials and Methods

This systematic review of the literature was conducted according to a prespecified protocol established at the inception of the study and reported in the light of the Preferred Reporting Items for Systematic Reviews and Meta-analyses (PRISMA) statement [31].

### 2.1. Eligibility Criteria

The research process and the selection of the eligible studies were based on the following PICOS criteria: P (population): The population was adults with a histopathologically confirmed diagnosis of synchronous or metachronous liver metastases from CRC. All metastatic disease had to be deemed potentially completely resectable (or in cases of combined treatment with surgery and radiofrequency ablation (RFA) or microwave ablation (MWA) procedure amenable to complete destruction) with macroscopically negative surgical margins while maintaining an adequate functional liver reserve. The patients with inoperable metastatic disease or solely extrahepatic lesions were excluded. I (Interventions): The treatment of the aforementioned patients, except for surgical resection of the primary tumor and liver metastasectomy, included either systemic chemotherapy with oxaliplatin-based or irinotecan-based regimens, fluoropyrimidines, systemic immunotherapy or monoclonal antibodies, or regional chemotherapy (HAI), or the combination of the two strategies. C (comparison): The therapeutic methods were compared either with no further intervention (surgery only) or among each other. O (outcomes): The prespecified outcomes were recurrence-free survival (RFS), disease-free survival (DFS), overall survival (OS) and severe adverse events. RFS was defined as the time interval from metastasectomy (or in the case of randomized clinical trials (RCTs) from randomization) to disease recurrence, death from disease, or the last follow-up date, while DFS was the length of time from liver resection (or in case of RCTs from randomization) to disease relapse, progression, or death due to cancer. OS was defined as the time from resection of liver lesions (or in the case of RCTs from randomization) until the date of death from any cause. Severe adverse events were defined as any grade 3 or 4 toxicities according to the WHO or the National Cancer Institute Common Toxicity Criteria classification described in each study. S (study design): Randomized clinical trials, non-randomized controlled studies, and single-arm trials along with prospective or retrospective cohort studies were deemed eligible for inclusion.

Searches were restricted to adult human subjects and English literature and were not limited by the date of publication. Furthermore, articles that did not contain original data (e.g., editorials, comments, reviews) along with pooled analyses and case reports were not included. Publications that did not contain RFS, DFS or OS outcomes or pertained to neoadjuvant therapy or unresectable liver metastases were also excluded.

### 2.2. Search Strategy

The database search was conducted using the following online resources: Medline/PubMed and the Cochrane Library from conception until 27 July 2022. “Snow-balling” was also performed by searching the references of included studies to minimize the possibility of article losses. Search terms were grouped as follows: group 1 (“Colorectal cancer liver metastases” OR “colorectal liver metastases” OR “metastatic colorectal cancer”); group 2 (hepatectomy OR metastasectomy OR “curative liver resection”); group 3 (“adjuvant therapy” OR “adjuvant chemotherapy” OR “systemic chemotherapy” OR “postoperative chemotherapy” OR “regional chemotherapy” OR “hepatic artery infusion”). Search results were extracted from the combination of groups 1, 2 and 3.

### 2.3. Study Selection and Data Abstraction

The research was conducted by EG and INS in accordance with the prespecified criteria, and relevant studies were identified by reviewing the titles and, when necessary, abstracts. After title and abstract eligibility screening, full-text assessment and data extraction from eligible trials were performed independently by two reviewers (EG and INS). Disagreements were resolved by consensus or the decision of a third reviewer (MG).

Extracted data items included the following: study year, study setting, study population size, median age of participants, number of patients with simultaneous resection for primary CRC and synchronous CRLMs, number of R0 vs. R1 resections of CRLMs, patients with synchronous vs. metachronous CRLMs, number of cases with lymph nodes metastases (LNMs) associated with the primary tumor, the median number of metastatic lesions, the median diameter of CRLMs, KRAS/BRAF status, CEA levels, median follow-up period, type of systematic chemotherapy, type of HAI regimen, RFS, DFS, OS and grade 3–4 complications from chemotherapy.

### 2.4. Risk of Bias Assessment

To assess the risk of bias, the following tools were used: (1) Revised Cochrane Risk of Bias Assessment 2 (RoB 2) tool for the randomized clinical trials, (2) the Risk of Bias in Non-randomized Studies (ROBINS-I) for the non-randomized trials and (3) the Newcastle–Ottawa scale for the retrospective cohort studies and the sole case–control study. The assessment was performed by two reviewers (EG and PM) independently. Disagreements were resolved by consensus or the decision of a third reviewer (DIT).

## 3. Results

### 3.1. Selection of Studies 

**Figure 1:**
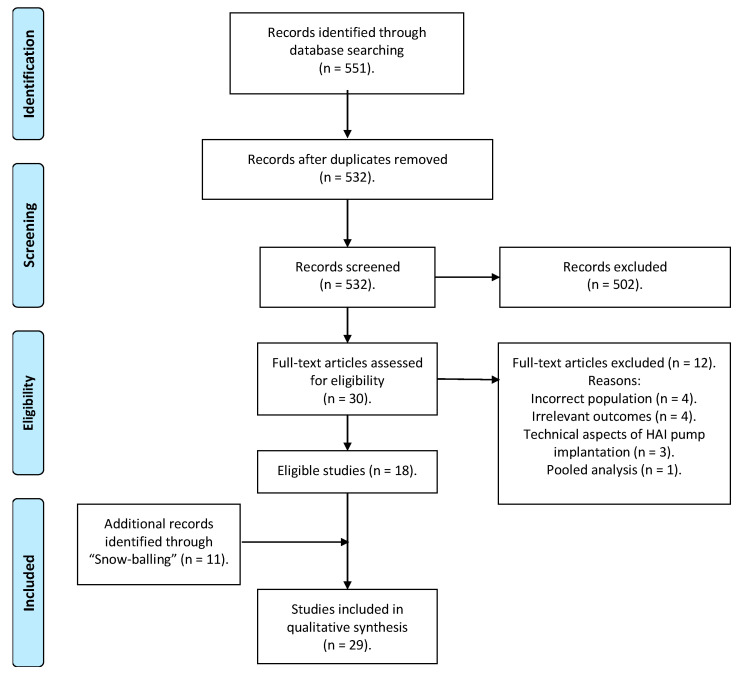
PRISMA flow diagram.

The research returned 551 results on 27 July 2022, 513 from PubMed and 38 from the Cochrane Library, leaving 532 articles eligible for screening after the removal of duplicates (Figure 1). Of the aforementioned publications, 500 were excluded as irrelevant according to their title and when necessary to their abstract, while two articles could not be retrieved. Twelve out of the thirty publications that were full-text assessed were deemed ineligible for the following reasons: incorrect population (one study included patients with unresectable CRLMs, one study included patients with extrahepatic CRC metastases and two studies included patients who received pre-operative chemotherapy), irrelevant outcomes (n = 4), publications that have to do exclusively with the technical aspects of the HAI pump implantation (n = 3) and one pooled analysis. Furthermore, 11 more articles from the “snowball” search were added to the 18 publications which fulfilled the inclusion criteria. Overall, a total of 29 studies were included, of which 9 were randomized clinical trials, 1 was a non-randomized controlled clinical study, 5 were phase II clinical trials, 2 were single-arm studies (in one of them the included patients were compared with historical controls) and 12 were retrospective cohort studies.

### 3.2. Patients Characteristics 

A total of 7028 patients were included in the eligible studies. They were adults with histologically proven CRLMs and no extrahepatic lesions who underwent therapeutic metastasectomy. In randomized clinical trials, there were 1541 participants who fulfilled the additional inclusion criteria of good performance status (ECOG 0-2) and adequate bone marrow, liver and kidney function. Furthermore, the patients who received HAI chemotherapy had undergone cholecystectomy and gastroduodenal devascularization as part of the HAI catheter or pump device placement process, in order to eliminate the risk of complications such as chemotherapy-related cholecystitis or gastritis, duodenitis and gastrointestinal ulceration due to drug misperfusion [32]. Synchronous metastases were detected in 2334 cases, while 266 of them underwent simultaneous curative resection for the primary lesion and CRLMs. Moreover, there were 4026 recorded R0 resections of CRLMs, whereas 3477 patients had lymph node metastases associated with the primary tumor. Regarding the rest of the extracted data, carcinoembryonic antigen (CEA) levels were available for 5693 subjects and KRAS status was available for 747 subjects. Moreover, 5797 patients received systemic chemotherapy, 122 received the anti-vascular endothelial growth factor (VEGF) monoclonal antibody bevacizumab, 14 received immunotherapy with tumor-infiltrating lymphocytes (TILs) and interleukin (IL)-2, 1478 underwent HAI of chemotherapy and 1204 underwent only surgical management without adjuvant treatment. Patient characteristics in studies including systemic and HAI chemotherapy are provided in Table 1 and Table 2, respectively.

### 3.3. Survival Outcomes

#### 3.3.1. Post-Metastasectomy Adjuvant Systemic Chemotherapy

The role of the fluoropyrimidine-based schemes was investigated by two randomized phase III studies. The first one was a multicenter randomized clinical trial that was conducted between December 1991 and December 2001 by Portier et al. [48]. A total of 171 patients from 47 hospitals in France and Switzerland were randomized after therapeutic metastasectomy of CRLMs either to receive 5-FU/LV or to serve as controls without adjuvant chemotherapy. The investigators recorded an improvement in DFS for patients in the chemotherapy group compared with the control group, but no statistically significant difference regarding OS. The 5-year DFS, after adjustment for major prognostic factors, was 33.5% vs. 26.7%, respectively (OR = 0.66; 95% CI, 0.46 to 0.96; *p* = 0.028), whereas the 5-year OS was 51.1% vs. 41.9%, respectively (OR = 0.73; 95% CI, 0.48 to 1.10; *p* = 0.13). The trial stopped due to a slow accrual rate. The second one took place in Japan from January 2004 through December 2010. In this multicenter open-label trial, 180 patients from 11 hospitals were enrolled, and the investigators compared surgery alone vs. metastasectomy followed by systemic oral uracil/tegafur with leucovorin. According to Kokudo et al. [34] following an extended follow-up period of 7.36 years, the RFS was significantly longer in the UFT/LV group compared to the surgery alone group (HR = 0.57; 95% CI, 0.39–0.84; *p* = 0.004), while OS did not differ significantly between the groups (HR = 0.86; 95% CI, 0.54–1.38; *p* = 0.54). Due to an insufficient number of events, the measurement of median OS was not feasible. 

Two further randomized clinical trials tested the role of the modern oxaliplatin- and irinotecan-based regimens. Kanemitsu et al. [33] evaluated the efficacy and safety of FOLFOX6 after hepatectomy vs. surgery-alone treatment. In this phase II/III controlled trial, 300 patients from Japan participated between March 2007 and January 2019, and the DFS was significantly better in the FOLFOX6 group in comparison with the monotherapy group. The 5-year DFS was 49.8% and 38.7%, respectively (HR = 0.67; 95% CI, 0.50–0.92; one-sided *p* = 0.006). However this progress did not seem to be correlated with better OS, since 5-year OS was inferior (71.2% and 83.1%, respectively; HR, 1.25; 95% CI, 0.78–2.00; two-sided *p* = 0.42). Ychou et al. [46] studied 306 patients from 66 centers across 15 countries from December 2001 through July 2006; the patients were randomized to receive either FOLFIRI or 5-FU/LV regimen. Although the investigators recorded a median DFS of 24.7 months in the FOLFIRI group vs. 21.6 months in the 5-FU/LV group, this difference was not statistically significant (HR, 0.89; 95% CI, 0.66–1.19; *p* = 0.44), whereas the use of FOLFIRI did not affect the OS. The 3-year survival rate was 72% for patients treated with 5-FU/LV and 73% for those receiving FOLFIRI (the median OS was not achieved). 

In a retrospective cohort study, Kim et al. [47] investigated the clinical outcomes of 156 cases treated with different chemotherapeutic agents after surgical resection of CRLMs. The patients were grouped regarding their adjuvant therapy as follows: oxaliplatin-based (group 1), irinotecan-based (group 2) and fluoropyrimidines alone (group 3). According to their results, oxaliplatin-treated patients (group 1) had significantly longer DFS in comparison with the other groups combined (median DFS 23.4 months vs. 14.9 months, respectively, *p* = 0.03), but there was not a significant difference in OS among the three groups. The median DFS was 23.4 months in group 1, 14.1 months in group 2 and 16.3 months in group 3 (*p* = 0.088). Conversely, Liu et al. [45], in a retrospective study of 52 patients with metachronous CRLMs, demonstrated that FOLFOX/FOLFIRI chemotherapy contributed to statistically significant amelioration of both DFS (HR = 0.37; 95% CI, 0.15–0.94; *p* = 0.036) and OS (HR = 0.27; 95% CI, 0.083–0.86; *p* = 0.026) in comparison with 5-FU/LV therapy.

Four retrospective studies explored the effectiveness of systematic chemotherapy in the adjuvant setting focusing on the timing of metastatic spread. In the most recent one, Sugimoto et al. [36] collated the efficacy of oxaliplatin-based schemes vs. fluoropyrimidine regimens among 94 subjects who had simultaneous surgical resection for colorectal cancer and synchronous CRLMs. After a median observation period of 64.5 months, they showed that RFS was significantly longer for patients in the oxaliplatin-based group in comparison with the ones in the fluoropyrimidine-based group in the propensity-matched cohort (HR = 0.40; 95% CI, 0.017–0.96; *p* = 0.04), but not in the overall cohort (HR = 0.80; 95% CI, 0.48–1.32; *p* = 0.38).

Hsu et al. [40] conducted a study with 72 patients with synchronous CRLMs; the patients were divided into three groups according to the adjuvant chemotherapy they received (group 1: 5-FU/LV, group 2: FOLFIRI/IFL, group 3: FOLFOX). The authors reported that patients treated with FOLFIRI in comparison with patients treated with 5-FU/LV had significantly better RFS (HR = 0.421; 95% CI, 0.209–0.847; *p* = 0.015) and OS (HR = 0.190; 95% CI, 0.068–0.527; *p* = 0.001), whereas patients treated with FOLFOX had only significantly better RFS than patients in group 1 (HR = 0.477; 95% CI, 0.230–0.988; *p* = 0.046) but without better OS (HR = 0.365; 95% CI, 0.119–1.119; *p* = 0.078) in the multivariate analysis. Nishioka et al. [38] demonstrated that adjuvant chemotherapy with either UFT/LV or oxaliplatin-based regimen compared with surgery alone significantly improved both the 5-year RFS and 5-year OS in patients with synchronous CRLMs (32.8% vs. 11.2%, *p* = 0.002, and 77.9% vs. 44.5%, *p* = 0.021, respectively) and early metachronous (≤12 months) CRLMs (43.7% vs. 15.2%, *p* = 0.002 and 81.5% vs. 39.5%, *p* = 0.015, respectively). However, there was no impact on the patients with late metachronous (>12 months) CRLMs, because 5-year DFS was 44.1% vs. 29.6% (*p* = 0.163) and the 5-year OS 76.1% vs. 65.4% (*p* = 0.411), respectively. The largest one including 1145 patients was conducted by Kobayashi et al. [37]. The investigators discriminated the subjects into two groups, namely patients who received adjuvant chemotherapy (group 1) and patients with surgical monotherapy (group 2), and showed that adjuvant chemotherapy in general was significantly beneficial in terms of RFS and OS for patients in the overall (RFS HR = 0.784, *p* = 0.045; OS HR = 0.716, *p* = 0.028) and synchronous cohorts (RFS HR = 0.677, *p* = 0.027; OS HR = 0.642, *p* = 0.036), but not for those in the metachronous cohort (RFS HR = 0.875, *p* = 0.378; OS HR = 0.881, *p* = 0.496). 

The benefit of oxaliplatin-based chemotherapy was suggested also by Sakamoto et al. [42] in a small study of 24 patients treated with FOLFOX4 or modified FOLFOX6 along with a single-arm phase II study by Satake et al. [35] that investigated the efficacy of CAPOX in 28 patients. The favorable role of FOLFOX and oxaliplatin plus 5-FU was proved by Kim et al. [44] in a study with sixty patients that were compared with historical controls. Mackay et al. [49] evaluated the role of irinotecan in another single-arm phase II clinical trial with 29 patients. The results were promising and support a further evaluation of irinotecan-based adjuvant chemotherapy after liver metastasectomy of CRM. The role of a 12-month S-1 scheme, an oral combination of tegafur and two enzyme inhibitors (gimeracil and oteracil), as an alternative approach after metastasectomy was explored by Kato et al. [39] in a single-arm phase II clinical trial with 62 patients from 19 hospitals. The study showed promising results in patients with low tumor burden, but no added benefit was noted regarding the patients in the high-risk group for recurrence, namely those with lymph node metastases around the primary site and/or early liver metastasis. 

Moreover, the role of bevacizumab in the adjuvant setting after CRLM resection was investigated by two studies. In a retrospective analysis by Turan et al. [41] of 204 patients that were treated with fluoropyrimidine-based, irinotecan-based and oxaliplatin-based regimens with or without bevacizumab, no significant differences were found in the median RFS (*p* = 0.375) and OS (*p* = 0.251) upon the addition of bevacizumab to chemotherapy. There was also no difference among the administered chemotherapy regimens regarding the median RFS (*p* = 0.744) and OS (*p* = 0.440). These findings were aligned with the results of a phase II randomized clinical trial with 73 patients conducted by Kemeny et al. [43]. Bevacizumab contributed neither to better RFS (4-year RFS in the bevacizumab arm was 46% vs. 37% in the control arm, *p* = 0.4) nor to better OS (4-year survival was 85% vs. 81%, respectively; *p* = 0.5). On top of these results, its use was also correlated with high rates of side effects, especially biliary toxicity.

Finally, Gardini et al. [50] tested the effectiveness of IL-2 in a non-randomized study with 36 patients, but the results showed that there were no significant differences in the actuarial and DFS rates between the group of patients who received the immunotherapy treatment and the control group. Table 3 summarizes the main survival outcomes of studies on adjuvant systemic therapy after CRLM resection.

#### 3.3.2. Post-Metastasectomy Adjuvant Hepatic Artery Infusion Chemotherapy

In a phase III clinical trial published in 1999, Kemeny et al. [58] randomized 156 patients who underwent surgical resection of CRLMs to receive either HAI-FUDR plus systemic chemotherapy (5-FU/LV) or systemic chemotherapy alone. The median OS was 72.2 months in the HAI group vs. 59.3 months in the monotherapy group, and the actuarial survival rates at two years were 86% vs. 72%, respectively (*p* = 0.03). In another randomized study between August 1990 and January 1997, the authors explored the efficacy of HAI-FUDR plus systemic 5-FU vs. surgical treatment alone [56]. The investigators showed that despite the significantly longer 4-year RFS for the chemotherapy-treated patients (46% in chemotherapy group vs. 25% in control group, *p* = 0.04), there was no impact on the OS (median 63.5 months in chemotherapy group vs. 49 months in control group, *p* = 0.60). Lorenz et al. [60] tried a different approach in a multicenter randomized phase III trial of 219 patients from April 1991 through December 1996 and compared HAI of 5-FU plus folinic acid with surgical monotherapy. They demonstrated that this type of HAI did not significantly improve survival and resulted in high rates of severe and potentially lethal toxicities. Therefore, the trial suspended patient accrual at the interim analysis due to futility. The reported median survival was 34.5 months for the HAI group versus 40.8 months for the control group (*p* = 0.1519). The efficacy of HAI with 5-FU was also investigated in a small randomized study in Japan by Tono et al. [57] from February 1993 to March 1995. The investigators compared 9 subjects treated with HAI to 10 patients treated with oral 5-FU only (the patients in the HAI group received also oral 5-FU after the completion of HAI therapy). Patients in the HAI group had statistically longer 3-year RFS (66.7% vs. 20%, *p* = 0.045), but this progress did not contribute to significantly longer survival (5-year survival rates: 77.8% vs. 50%, respectively, *p* = 0.2686). In addition, the beneficial role of HAI-FUDR plus systemic chemotherapy with oxaliplatin and capecitabine was suggested by Alberts et al. [55] in a single-arm phase II clinical study of 55 patients. According to their results, this combination met the prespecified endpoint of higher than 85% survival at two years and was clinically tolerable. 

The role of HAI was further investigated by four retrospective cohort studies. The largest one by Buisman et al. [52] included 2128 patients from the databases of two centers (MSKCC and Erasmus MC Cancer Institute), who received either HAI-FUDR plus systemic chemotherapy treatment or systemic chemotherapy alone. According to the investigators, HAI contributed to significantly better DFS (adjusted HR = 0.69; 95% CI, 0.60–0.79; *p* < 0.001) and OS (adjusted HR = 0.67; 95% CI, 0.57–0.78; *p* < 0.001). The same approach was chosen by House et al. [54] in a study with 250 patients from MSKCC comparing systemic chemotherapy (5-FU/LV plus oxaliplatin or irinotecan) with or without HAI-FUDR after metastasectomy. The findings showed that the 5-year RFS was significantly better for the HAI plus systemic therapy group than for the patients who received only systemic therapy (48% vs. 25%, respectively, *p* < 0.01). On the other hand, Goéré et al. [53] collated the outcomes of 44 patients treated postoperatively with HAI of oxaliplatin plus systemic 5-FU with those of 54 patients who received systemic chemotherapy alone (FOLFOX or FOLFIRI). Although there was a significant prolongation of the 3-year DFS in the HAI group (33% vs. 5%, respectively, *p* < 0.0001), the 3-year OS was not significantly improved (75% vs. 62%, respectively, *p* = 0.17). 

Furthermore, Gholami et al. [51] reported on KRAS mutational status in a retrospective cohort study of 674 patients who received either HAI-FUDR plus systemic chemotherapy or only systemic chemotherapy and showed that it did not influence the outcomes. The 5-year OS was 78% in the HAI group vs. 57% in the no-HAI group (HR = 0.51, *p* < 0.001) in patients with KRAS wild type and 59% in the HAI group vs. 40% in the no-HAI group (HR = 0.56, *p* < 0.001) in patients with mutations in KRAS.

In addition, the role of mitomycin C plus 5-FU as an alternative HAI scheme was explored in two studies. Rudroff et al. [59], in a randomized study with 42 patients, showed that this treatment added no benefit compared with surgery alone regarding both DFS and OS. Kokudo et al. [61], in a retrospective study with 115 patients, compared the efficacy of oral (UTF/5-DFUR) or IV (MMC or 5-FU) systemic chemotherapy (group 1) to that of intra-arterial/intraportal regional therapy (5-FU/LV or MMC/5-FU/doxorubicin) (group 2) or surgery alone (group 3). The authors found a significant improvement in DFS (5-year DFS: 33%, 26% and 19%, respectively; *p* = 0.02) but not in OS (5-year OS: 51%, 49% and 37%, respectively; *p* = 0.37). Table 4 summarizes the survival outcomes of studies on adjuvant HAI therapy following CRLM resection.

### 3.4. Side Effects

#### 3.4.1. Post-Metastasectomy Adjuvant Systemic Chemotherapy

Safety data were available for 1908 subjects in 15 of the chosen studies. Portier et al. [48] demonstrated that 54 out of 81 finally treated participants (54%) in the chemotherapy group had a complete course, which was defined as more than 85% of the planned dose. Severe adverse events according to WHO classification were reported in 20 patients (24.7%), the most frequent of which were diarrhea (n = 7), hematologic events (n = 6), stomatitis (n = 6), nausea (n = 6) and neuropathy (n = 2), and 12 of them manifested more than one. Kanemitsu et al. [33] were forced to suspend enrollment after the first 2 years of their trial because of high rates of toxicities. After the necessary protocol changes (higher WBC count in the inclusion criteria, lower dose and intensity of chemotherapy) the rate of adherence and tolerance was higher. The most common severe side effects were neutropenia, reported in 50% of patients, along with sensory neuropathy (10%) and allergy (4%).

In reported feasibility and safety data, Saiura et al. [62] stated that among the 82 subjects in the UFT/LV arm who were included in the safety analysis, 12.2% (n = 10) had adverse events of grade 3 or higher. The most common were hematologic events (anemia 3.7%, febrile neutropenia 1.2%) and diarrhea (4.9%). Moreover, Nishioka et al. [38] reported that severe toxicities were significantly more frequent in the oxaliplatin group (50.9%) compared to the UFT group (6.8%, *p* < 0.001), which led to higher adherence in the UFT arm (84.1%, *p* < 0.001). Neutropenia was the most common hematological toxicity (5%) also among the patients who received the S-1 regimen according to the results of Kato et al. [39]. The most frequent one though was fatigue in 6.7% of cases.

Ychou et al. [46] demonstrated that adherence did not differ significantly between the two chemotherapy groups. In general, the patients in the 5-FU/LV group received higher cumulative doses, and the patients in the FOLFIRI group had more cycles with dose reductions than the ones in the 5-FU/LV group. Grade 3 or 4 treatment-related adverse events were reported in 30% of patients receiving 5-FU/LV vs. 47% of patients receiving FOLFIRI. Neutropenia and diarrhea were once more the most common severe toxicities in both treatment arms, especially in the FOLFIRI group (23% vs. 7% and 14% vs. 7%, respectively). The association of irinotecan with high rates of grade 3 or 4 neutropenia (20.7%) was shown by Mackay et al. [49], along with severe diarrhea (17.2%) and grade 3 vomiting (13.2%). 

In the study of Satake et al. [35], among 28 participants, 20 (71.4%, 95% CI: 53.6–89.3%) were able to complete the protocol treatment. Capecitabine was reduced in 10 (36%) and discontinued in 5 subjects (19%), whereas oxaliplatin dose reduction was reported in eight cases (29%) and discontinued in eight subjects (29%). The most common high-grade toxicity was neutropenia (29%). Furthermore, the correlation between capecitabine and high rates of toxicities was reported by Alberts et al. [55]. According to the investigators, 32 out of 55 participants experienced at least one grade 3 adverse event, the most frequent of which were gastrointestinal toxicities and paresthesia.

Regarding the use of bevacizumab, the phase II study investigating its role in the adjuvant setting was terminated early due to the high incidence of biliary toxicity in the bevacizumab arm (14% of patients in the bevacizumab arm suffered from a bilirubin elevation >3 mg/dL, *p* = 0.02, and the placement of biliary stents was deemed necessary in 11% of them) [43]. A completely different pattern of toxicities was shown by Gardini et al. [50]. The investigators recorded mild epileptic fits (probably due to hyperpyrexia), renal dysfunction with diuresis <200 mL/12 h, prolonged tachycardia and fever >39C, which led to temporary discontinuation of treatment in three patients (21%) and resolved after treatment suspension. Table 5 summarizes the main severe toxicities reported by the eligible studies. 

#### 3.4.2. Post-Metastasectomy Adjuvant Hepatic Artery Infusion Chemotherapy

The toxic effects of systemic chemotherapy were similar in both treatment arms in the study of Kemeny et al. On the contrary, the tripling of serum aspartate aminotransferase levels (65%), the doubling of the serum alkaline phosphatase level (29%), and the increase in total serum bilirubin levels to more than 3.0 mg/dL (18%) in subjects that received the dual therapy were attributed solely to HAI chemotherapy. Moreover, four of them required biliary stents [58]. The same pattern of toxicities was also revealed in the comparison of HAI plus systemic chemotherapy vs. surgery alone [56]. Out of the 30 patients in the combined therapy arm, 9 experienced a grade 3 increase in liver enzymes and 2 of them developed biliary sclerosis; thus, bile duct stenting was deemed necessary. The decrease in dosage or the discontinuation of HAI chemotherapy led to the normalization of liver enzymes in the rest of the cases. However, no fatal side effects were recorded in this group. Moreover, in the study by Alberts et al. [55], the HAI therapy seemed to be related to regional side effects (e.g., catheter occlusion or pump malfunction) rather than systemic toxicities.

Regarding the alternative HAI regimens, high rates of severe toxicities were recorded by Lorenz et al. [60]. Forty-four (62.9%) out of seventy-three patients, for whom there were available toxicity data, experienced grade 3 or 4 toxicity during a total of 76 cycles of treatment (25.6%). The most frequent ones were stomatitis, nausea/vomiting, pain and diarrhea. Conversely, Tono et al. [57] reported no severe toxicities, but the sample of patients who received HAI was small.

### 3.5. Risk of Bias Assessment

According to the RoB 2 tool for the randomized clinical trials, the ROBINS-I for the non-randomized trials and the Newcastle–Ottawa scale for the retrospective cohort studies and the case–control study, the eligible studies were evaluated as follows:

- Five randomized studies of low [57] or moderate risk [33,34,43,60] and five of high risk [46,48,56,58,59] (Appendix A Table A1).
- One non-randomized clinical study in which the risk of bias was judged as serious [50] (Appendix A Table A2).
- Ten cohort studies of good [36,37,40,41,45,47,51,52,53,54] and two of poor quality [38,61] (Appendix A Table A3).
- The case–control study was considered as poor quality [44] (Appendix A Table A4).

## 4. Discussion

Our goal was to collate the currently available data from studies investigating the role of adjuvant therapy in patients with resected CRLMs, aiming to provide insight into the efficacy as well as the safety of the multiple regimens in the clinical practice. The three randomized clinical trials, which compared adjuvant systemic chemotherapy to surgical monotherapy, failed to show a significant difference in terms of OS, regardless of the statistically significant prolongation of DFS or RFS, after correction for prognostic factors. These results are in accordance with former reviews, randomized clinical trials or pooled analyses [63,64], despite the fact that they also included patients who received perioperative chemotherapy or had extrahepatic CRM. To explain the discrepancy between DFS/RFS and OS, we considered the following possible etiologies.

Firstly, there was high heterogeneity regarding the basic characteristics of patients included in these studies, such as the timing of metastases and the number of CRLMs. This rationale was confirmed by the two retrospective studies, which explored the role of adjuvant chemotherapy in specific populations. Kobayashi et al. [37] demonstrated statistically significant improvement of both RFS and OS in patients with synchronous metastases, but not for those with metachronous ones. Nishioka et al. [38] showed the same results not only in patients with synchronous metastatic disease, but also in patients with early-metachronous (<12 months) metastatic disease. The most frequently used agents in these studies were oxaliplatin-based schemes and UFT/LV.

Moreover, in contrast to patients who had only metastasectomy, those who received adjuvant chemotherapy after surgery experienced more severe adverse events and had lower adherence to treatment. Oxaliplatin is associated with sinusoidal obstruction syndrome (SOS), nodular regenerative hyperplasia (NRH) [65] and steatohepatitis, which makes the visualization of recurrent lesions on CT difficult because of the heterogeneity of liver parenchyma [66]. SOS is defined as the non-thrombotic obstruction of sinusoids by damaged hepatic sinusoidal endothelial cells that could also be provoked after radiation therapy or high-dose anticancer drugs given before a stem cell transplant, which causes liver damage. Furthermore, adjuvant chemotherapy could have successfully treated only the chemosensitive tumor cells and induced resistance in the remaining micro-metastases (selective pressure) due to an adjuvant therapy-related shortening of survival (ATRESS) phenomenon. This phenomenon is secondary to preselection inherent in adjuvant therapy, and it leads to the elimination of the less malignant tumor cells, sparing the most aggressive tumor clones. Thus, re-exposure to chemotherapeutic agents is less effective [67]. Another possible explanation is that adjuvant therapy can delay the appearance but not eradicate micro-metastases that already exist at the time of liver resection.

Many studies compared the modern oxaliplatin- and irinotecan-based regimens to the traditional fluoropyrimidine schemes, but still the results are unclear. In general, oxaliplatin- and irinotecan-based regimens contributed to significantly better RFS/DFS. However, a significant prolongation in OS was recorded only by Hsu et al. [40] with the FOLFIRI regimen in patients with synchronous disease and by Liu et al. [45] with FOLFOX/FOLFIRI in patients with metachronous disease. On the other hand, the comparison of a cohort with sixty patients treated with oxaliplatin-based chemotherapy to historical controls who received 5-FU/LV by Kim et al. [44] showed no better RFS or OS, while Ychou et al. [46] in a clinical trial demonstrated that the FOLFIRI regimen did not contribute to a significant amelioration in DFS and OS in comparison with 5-FU/LV, while it was less tolerable. Bevacizumab was evaluated in a randomized clinical trial and a retrospective study that found low efficacy and an increase in severe toxicities. The results of the Hepatica study, which is a two-arm multicenter randomized clinical trial that investigates the efficacy of bevacizumab in addition to adjuvant therapy with CAPOX, are anticipated. In the end, the results of immunotherapy with TIL+IL2 were not promising and provided little support for the addition of this therapeutic approach in the adjuvant setting after liver metastasectomy [50].

Regarding the efficacy of HAI chemotherapy, statistically significant survival was recorded only in one randomized trial and the two largest retrospective studies. The rest of the chosen studies failed to confirm this survival benefit, despite the significantly improved RFS/DFS rates. Moreover, Gholami et al. [51] showed a survival benefit regardless of the KRAS status, while Buisman et al. [52] proved that patients treated with HAI had a significantly higher incidence of extrahepatic recurrences, mainly pulmonary, which contributed to similar recurrence rate between the two groups, but with better DFS. This finding may reaffirm the necessity of a systemic approach to CRLMs, because HAI may suppress the recurrence only to the residual liver. The most investigated regimen was FUDR in combination with dexamethasone to eliminate HAI-related side effects, but the alternative approaches also failed to improve the outcomes; specifically, the use of 5–fluorouracil and folinic acid was correlated with high rates of toxicities. There are two further ongoing clinical trials evaluating the efficacy of HAI chemotherapy. The first one compares HAI-FUDR to surgery-alone treatment in patients with resectable CRLMs of low risk (PUMP trial; Netherlands Trial Register (NTR) number: 7493), while the second one compares systemic FOLFOX to HAI treatment with oxaliplatin plus systemic 5-FU/LV in patients with at least four CRLMs (PACHA-01; ClinicalTrials.gov NCT02494973).

Before the addition of HAI chemotherapy after the resection of CRLMs, the difficulties of this method may also be taken into consideration. Its implantation has a learning curve, and only a highly trained medical team in specified centers should undertake this procedure; otherwise, there is an increased risk of failure or pump/catheter-related complications. Moreover, its high incidence of toxicities may undermine adherence to the therapeutic scheme.

This review has several limitations. First, the literature research was conducted only in the English language, which makes the omission of some non-English studies possible. Secondly, the majority of the chosen randomized clinical trials were assessed as being of modest or high risk, a fact suggesting that the results of this review could have been biased and subject to residual confounding. Moreover, there were discrepancies among the included studies regarding the definitions of outcomes of interest. In the non-randomized studies, DFS, RFS and OS were calculated from the time of hepatic resection, while in many RCTs DFS, RFS and OS were calculated from the time of randomization. In the case of RCTs that studied the role of HAI [57,58,59,60], the patients were randomized intraoperatively, while in the case of some RCTs that investigated the role of systemic adjuvant therapy, the randomization process took place after the surgery [33,34]. Furthermore, overlaps of patients between the studies of Gholami et al. [51] and Buisman et al. [52] could be possible since many of the included patients were treated at the same center (MSKCC) in a similar period, although the two studies had distinct authorship. Finally yet importantly, we did not perform a meta-analysis due to the significant heterogeneity in terms of patient and treatment characteristics among the eligible studies. 

### Future Directions

Taking the results of the included studies in this systematic review under consideration, there is still a need for more randomized clinical trials that compare the efficacy, the safety and the tolerability of chemotherapeutic agents. The comparison between chemotherapy and surgical monotherapy, although very informative, could also be problematic because the eligible patients and their families may be reluctant to participate in such trials when they consider the possibility of being assigned to surgery-alone treatment. Aside from the traditional IV schemes such as FOLFOX, FOLFIRI and 5-FU/LV, the oral alternatives such as CAPOX or UFT/LV, which are gaining ground in the modern therapeutic armamentarium, should also be further evaluated due to their advantages such as better tolerance and compliance. The role of targeted treatments and immunotherapies such as checkpoint inhibitors should also be evaluated [68,69]. Novel treatment combinations including immunotherapy may even lead to the disappearance of CRLMs upon imaging, which poses new questions in terms of the need for surgery and adjuvant therapy [70].

Of course, in an era that moves forward to individualized therapies, the discrimination between high- and low-risk patients on the basis of risk factors such as the timing of liver metastases would be crucial in order to find out which populations will benefit most from adjuvant therapy after liver metastasectomy. The two aforementioned ongoing trials regarding the efficacy of HAI chemotherapy follow this example. To that end, genetic mutation analysis may aid the stratification of patients and possibly guide the therapeutic approach [71]. For instance, it is well known that BRAF and KRAS mutations are considered negative prognostic markers [71,72]. However, since a prospective randomized clinical trial evaluating biomarkers in question may not always be feasible, the use of archived material from studies originally designed to investigate the efficacy of a treatment would be an acceptable alternative strategy, as these trials always include a relevant control group. 

In addition, it is expected that liquid biopsy, as an indicator of active disease, would be of great importance in the future. According to Reiner et al. [73], ctDNA-negative patients have a low risk of relapse. Therefore, it could be a feasible option for them to be actively surveilled instead of receiving adjuvant chemotherapy, thus sparing this population from the toxic effects of chemotherapy and the risk of the ATRESS phenomenon. The longitudinal ctDNA before, during and after adjuvant chemotherapy could also provide a patient-level measurement of its effectiveness [74]. Finally, the impact of various predictive biomarkers on already existing or new regimens should be further investigated either in parallel or genotype-driven (umbrella/basket trials) clinical trials. Their study requires not only the right assays (e.g., RT-PCR, FISH, IH) with predefined cut-off values and scoring methodologies, but also the right tissue sample, preferably from the metastatic lesions themselves and not the primary tumor. The modulation of their expression could also be a very interesting concept for the development of new targeted therapies. Examples of such biomarkers are the enzymes thymidylate synthase and dihydropyrimidine dehydrogenase, which inhibit the metabolism of 5-FU to its active metabolites intracellularly, and the excision repair cross-complementation group 1 enzyme, which is responsible for oxaliplatin resistance [75,76].

## 5. Conclusions

Despite the fact that the results of this systematic review may seem contradictory due to the high heterogeneity in patient populations and outcomes in the available studies, more high-quality clinical trials conducted on sub-populations with specific risk factors are required to determine the patient subgroups that will derive the optimal benefit from adjuvant therapy after CRLM resection.

## Figures and Tables

**Table 1:** Characteristics of patients in systemic chemotherapy studies.

Reference	Setting	Randomization	Regimens of Chemotherapy	Number of Patients	Age (Range)	Mean Follow-Up	Risk Factors
Kanemitsu et al., 2021 [33]	Phase II/III	YES	FOLFOX6 vs. SA	FOLFOX6 (n = 151), SA (n = 149)	FOLFOX6, 63 y (56–69); SA, 65 y (58–69)	59.2 mo (IQR 26.5–95.3)	Timing, number and diameter of CRLMs, R0 resections, LNM
Kokudo et al., 2021 [34]	Phase III	YES	UFT/LV vs. SA	UFT/LV (n = 90), SA (n = 90)	UFT/LV, 62.2 y (SD 8.5); SA, 64.5 y (SD 9.2)	7.36 y (6.93–7.87)	Timing, number and diameter of CRLMs, R0 resections, LNM
Satake et al., 2021 [35]	Phase II	NO	CAPOX	28	69.5 y (39–82)	64.3 mo (26.3–87.5)	Timing, number and diameter of CRLMs, R0 resections, LNM
Sugimoto et al., 2021 [36]	Retrospective	NO	Oxaliplatin-based vs. fluoropyrimidine regimen	Oxaliplatin-based (n = 47), fluoropyrimidine regimen (n = 47)	Oxaliplatin-based, 61 y (32–78); Fluoropyrimidine, 67 y (37–85)	64.5 mo (7.5–163.1)	Timing, number and diameter of CRLMs, R0 resections, LNM, CEA levels
Kobayashi et al., 2019 [37]	Retrospective	NO	AC (FOLFOX, FOLFIRI, CapeOx, 5-FU/LV, UFT/LV, S-1, HAI, doxifluridine) vs. SA	AC (n = 771), SA (n = 374)	AC < 65 y (n = 411); ≥65 y (n = 360); SA, <65 y (n = 145); ≥65 y (n = 229)	79.4 mo	Timing, number and diameter of CRLMs, R0 resections, LNM, CEA levels
Nishioka et al., 2017 [38]	Retrospective	NO	UFT/LV or oxaliplatin-based chemotherapy vs. SA	AC (n = 105), SA (n = 104)	AC, 65 y (36–88) SA: 63 y (35–74)	AC: 48 mo, SA: 28 mo	Timing, number and diameter of CRLMs, R0 resections, LNM, CEA levels
Kato et al., 2015 [39]	Phase II	NO	S-1	60	64 y (43–78)	41 mo (5–57)	Timing, number and diameter of CRLMs, R0 resections, LNM, CEA levels
Hsu et al., 2013 [40]	Retrospective	NO	5-FU/LV vs. FOLFIRI/IFL vs. FOLFOX	5-FU/LV (n = 25) FOLFIRI/IFL (n = 21), FOLFOX (n = 26)	58 y (26–76)	38.8 mo	Timing, number and diameter of CRLMs, R0 resections, CEA levels
Turan et al., 2013 [41]	Retrospective	NO	BEV vs. No BEV	BEV (n = 87), No BEV (n = 117)	56 y (24–82)	27 mo	Timing, number and diameter of CRLMs, R0 resections, LNM
Sakamoto et al., 2012 [42]	Single-arm	NO	FOLFOX4/modified FOLFOX6	24	58 yr (25–77)	48.4 mo	Timing, number and diameter of CRLMs, LNM
Kemeny et al., 2011 [43]	Phase II	YES	BEV vs. No BEV	BEV (n = 35), No BEV (n = 38)	Bev, ≥60 y (n = 10); <60 y (n = 25); No Bev, ≥60 y (n = 11) <60 y (n = 27)	30 mo	Timing, number and diameter of CRLMs, R0 resections, LNM, CEA levels, KRAS status
Kim et al., 2011 [44]	Retrospective	NO	FOLFOX4/modified FOLFOX6	60	55 y (31–73)	33.1 mo (95% CI: 4.1–108.5)	Timing, number and diameter of CRLMs, R0 resections, LNM, CEA levels
Liu et al., 2010 [45]	Retrospective	NO	FOLFOX/FOLFIRI vs. 5- FU/LV	FOLFOX/FOLFIRI (n = 31), 5-FU/LV (n = 19)	70 y (47–85)	35.5 mo (10–96)	Timing, number and diameter of CRLMs, R0 resections, CEA levels
Ychou et al., 2009 [46]	Phase III	YES	FOLFIRI vs. 5-FU/LV	FOLFIRI (n = 153), 5-FU/LV (n = 153)	FOLFIRI, 63 y (27–75); 5-FU/LV, 61 y (34–76)	FOLFIRI; 41.7 mo, LV5-FU; 42.4 mo	Timing and number of CRLMs, R0 resections,
Kim et al., 2009 [47]	Retrospective	NO	Oxaliplatin (group 1) vs. irinotecan (group 2) vs. fluoropyrimidine alone (group 3) regimens	group 1 (n = 58), group 2 (n = 48), group 3 (n = 50)	group 1, 61 y (25–78) group 2, 57 y (33–72) group 3, 61 y (32–77)	44 mo (18.4–86.9)	Number and diameter of CRLMs, R0 resections, LNM CEA levels
Portier et al., 2006 [48]	Phase III	YES	5-FU + folinic acid vs. SA	AC (n = 86), SA (n = 85)	AC, <55 y (n = 16); 55–64 y (n = 34); >65 y (n = 36) SA, <55 y (n = 15) 55–64 y (n = 37); >65 y (n = 33)	87.4 mo (SE = 5.8)	Timing, number and diameter of CRLMs, R0 resections, CEA levels
Mackay et al., 2005 [49]	Phase II	NO	Irinotecan	29	57 y (40–71)	27.9 mo (17.4–45.7)	Timing and number of CRLMs
Gardini et al., 2004 [50]	Clinical trial	NO	TIL+IL-2 vs. SA	TIL+IL-2 (n = 14), SA (n = 22)	TIL+IL-2, 57 y (40–70) SA, 57 y (37–70)	42 mo	Number and diameter of CRLMs, CEA levels

Abbreviations: 5-FU: 5-fluorouracil; 5-FU/LV: 5-fluorouracil/leucovorin; BEV: bevacizumab; CAPOX: capecitabine plus oxaliplatin; FOLFIRI: folinic acid, 5-FU plus irinotecan; FOLFOX: 5-FU/LV plus oxaliplatin; S-1: tegafur plus gimeracil and oteracil; TIL+IL2: tumor-infiltrating lymphocytes plus interleukin-2; UFT/LV: uracil/tegafur plus LV; AC: adjuvant chemotherapy; SA: surgery alone; CEA: carcinoembryonic antigen; LNM: lymph node metastasis; y: year; mo: months.

**Table 2:** Characteristics of patients in HAI chemotherapy studies.

Reference	Setting	Randomization	Regimens of Chemotherapy	Number of Patients	Age (Range)	Mean Follow-Up	Risk Factors
Gholami et al., 2020 [51]	Retrospective	NO	HAI (FUDR) vs. No HAI	HAI (n = 366), No HAI (n = 308)	HAI, 55 yr (47–63); No HAI, 62.5 yr (52–71)	6.5 yr	Timing, number and diameter of CRLMs, R0 resections, LNM, KRAS status
Buisman et al., 2020 [52]	Retrospective	NO	HAI (FUDR) + systemic chemotherapy vs. systemic chemotherapy alone	HAI (n = 601), No HAI (n = 1527)	HAI, 57.2 yr (IQR 49–65.5); No HAI, 63 yr (IQR 54.1–70.4)	96 mo (IQR 61–133)	Number and diameter of CRLMs, LNM, CEA levels
Goéré et al., 2013 [53]	Cohort study	NO	HAI (oxaliplatin) + systemic 5-FU/LV vs. systemic irinotecan or oxaliplatin regimes alone	HAI (n = 44), No HAI (n = 54)	HAI, 55 yr (47–63); No HAI, 58 yr (49–67)	60 mo (51–81)	Timing, number and diameter of CRLMs, R0 resections, LNM, CEA levels
House et al., 2011 [54]	Cohort study	NO	HAI (FUDR) + systemic chemotherapy (5-FU/LV + irinotecan or oxaliplatin) vs. systemic chemotherapy alone	HAI (n = 125), No HAI (n = 125)	HAI, 55 yr (28–80); No HAI, 61 yr (25–84)	43 mo (0.5–92)	Number and diameter of CRLMs, LNM, CEA levels
Alberts et al., 2010 [55]	Phase II	NO	HAI (FUDR) + systemic chemotherapy (oxaliplatin+capecitabine)	55	capecitabine 2000 mg/m²/d, 55 yr (34–79); capecitabine 1700 mg/m²/d, 60 yr (41–69)	4.8 yr	R0 resections
Kemeny et al., 2002 [56]	Phase III	YES	HAI (FUDR) + systemic chemotherapy (5-FU)	AC (n = 30), SA (n = 45)	AC, 59 yr (28–71); SA, 62 yr (29–78)	51 mo	Timing of CRLMs
Tono et al., 2000 [57]	Phase III	YES	HAI (5-FU) vs. oral 5-FU	HAI (n = 9), 5-FU (n = 10)	HAI: 59 yr (SD ± 5.8); control: 61.9 y (SD ± 5.0)	62.2 mo	Timing, number and diameter of CRLMs
Kemeny et al., 1999 [58]	Phase III	YES	HAI (FUDR) + systemic chemotherapy (5-FU + leucovorin) vs. systemic chemotherapy alone	HAI (n = 74), No HAI (n = 82)	HAI, 59 yr (28–79); No HAI, 59 yr (30–77)	62.7 mo (range 16–95)	Timing and number of CRLMs, R0 resections, CEA levels
Rudroff et al., 1999 [59]	Phase III	YES	HAI (mitomycin C/5-FU) vs. SA	Group A (n = 14), Group B (n = 16), Group C (n = 12)	Group A, 58 yr (39–70); Group B, 57 yr (45–76); Group C, 58 yr (46–79)	NR	Timing, number and diameter of CRLMs, LNM, CEA levels
Lorenz et al., 1998 [60]	Phase III	YES	HAI (5-FU/FA) vs. SA	HAI (n = 108), SA (n = 111)	61 yr (30–76)	At least 18 mo	Timing and number of CRLMs, LNM
Kokudo et al., 1998 [61]	Retrospective	NO	Group 1: oral (UTF/5-DFUR) or IV (MMC or 5-FU) systemic chemotherapy vs. group 2: intra-arterial/intraportal regional therapy (5-FU/LV or MMC/5-FU/doxorubicin) vs. group 3: no adjuvant therapy	Group 1 (n = 37) vs. group 2 (n = 38) vs. group 3 (n = 40)	60 yr (38–79)	NR	Number and diameter of CRLMs, LNM, CEA levels

Abbreviations: 5-FU: 5-fluorouracil; 5-FU/LV: 5-fluorouracil/leucovorin; 5-FU/FA: 5-fluorouracil plus folinic acid; FUDR: floxuridine; AC: adjuvant chemotherapy; SA: surgery alone; CEA: carcinoembryonic antigen; LNM: lymph node metastasis; NR: not reported; yr: year; mo: months.

**Table 3:** Outcomes of studies investigating systemic adjuvant systemic therapy.

Reference	Regimens of Chemotherapy	Outcomes		
		DFS	RFS	OS
Kanemitsu et al., 2021 [33]	FOLFOX6 vs. SA	5-yr DFS: 49.8% (95% CI, 41.0–58.0) vs. 38.7% (95% CI, 30.4–46.8), HR = 0.67 (95% CI, 0.50–0.92, *p* = 0.006)	-	5-yr OS: 71.2% (95% CI, 61.7–78.8) vs. 83.1% (95% CI, 74.9–88.9); HR = 1.25 (95% CI 0.78–2.00, *p* = 0.42)
Kokudo et al., 2021 [34]	UFT/LV vs. SA	-	HR = 0.57 (95% CI: 0.39–0.84, *p* = 0.004)	OS: HR = 0.86 (95% CI, 0.54–1.38, *p* = 0.54)
Satake et al., 2021 [35]	CAPOX	-	5-yr RFS 65.2% (95% CI: 46.48–83.92%)	5-yr OS: 87.2%
Sugimoto et al., 2021 [36]	Oxaliplatin-based vs. fluoropyrimidine regimen	-	RFS: HR = 0.80 (95% CI: 0.48–1.32, *p* = 0.38)	NR
Kobayashi et al., 2019 [37]	AC (FOLFOX, FOLFIRI, CapeOx, 5-FU/LV, UFT/LV, S-1, HAI, doxifluridine) vs. SA	-	5-yr RFS: 40.1% (33.4–46.7%) vs. 36.6% (30–43.3%), HR = 0.784 (95% CI 0.618–0.0995)	5-yr OS: 66.8% (95 CI 59.7–72.9%) vs. 59.6% (52.1–66.2%), HR = 0.716 (95% CI 0.532–0.964)
Nishioka et al., 2017 [38]	UFT/LV or oxaliplatin-based chemotherapy vs. SA	-	5-yr RFS: 32.8% vs. 11.2% in S-CLM (*p* = 0.002), 43.7% vs. 15.2% in EM-CLM (*p* = 0.002), 44.1% vs. 29.6% LM-CLM (*p* = 0.411)	5-yr OS: 77.9% vs. 44.5% in S-CLM (*p* = 0.021), 81.5% vs. 39.5% in EM-CLM (*p* = 0.015), 76.1% vs. 65.4% LM-CLM (*p* = 0.411)
Kato et al., 2015 [39]	S-1	3-yr DFS 47.4%	-	3-yr OS: 80.0%
Hsu et al., 2013 [40]	5-FU/LV vs. FOLFIRI/IFL vs. FOLFOX	-	Median RFS: 14.4, 20.8, 18.8 mo; 4-yr RFS HR (FOLFIRI vs. 5-FU/LV) = 0.421 (95% CI: 0.209-0.847, *p* = 0.015); HR (FOLFOX vs. 5-FU/LV) = 0.477 (95% CI: 0.230–0.988, *p* = 0.046)	5-yr OS 13%, 53% and 63%; HR (FOLFIRI vs. 5-FU/LV) = 0.190 (95% CI: 0.068–0.527, *p* = 0.001); HR (FOLFOX vs. 5-FU/LV) = 0.365 (95% CI: 0.119–1.119, *p* = 0.078)
Turan et al., 2013 [41]	BEV vs. No BEV	-	Median RFS: 14 vs. 18 mo (*p* = 0.375)	Median OS: 43 vs. 54 mo (*p* = 0.251)
Sakamoto et al., 2012 [42]	FOLFOX4/modified FOLFOX6	5-yr DFS: 45.1%	-	5-yr OS: 76%
Kemeny et al., 2011 [43]	BEV vs. No BEV	-	4-yr RFS 37% vs. 46%; *p* = 0.4	4-yr OS 81% vs. 85%, *p* = 0.5
Kim et al., 2011 [44]	FOLFOX4/modified FOLFOX6	-	5-yr RFS: 39.2%	5-yr OS: 55.5%
Liu et al., 2010 [45]	FOLFOX/FOLFIRI vs. FU/LV	3-yr DFS: 50.8% vs. 21.1%; HR = 0.37 (95% CI: 0.15–0.94, *p* = 0.022)	-	3-yr OS: 85.7% vs. 51.8% (*p* = 0.027); 5-yr OS: 54% vs. 34.6% (*p* = 0.027); HR = 0.27 (95% CI: 0.083–0.86)
Ychou et al., 2009 [46]	FOLFIRI vs. FU/LV	2-yr DFS 50.7% vs. 46.2%; HR = 0.89 (95% CI: 0.66–1.19, *p* = 0.44)	-	3-yr OS: 72.7% VS. 71.6%, HR = 1.09 (95% CI: 0.72–1.64, *p* = 0.69)
Kim et al., 2009 [47]	Oxaliplatin (group 1) vs. irinotecan (group 2) vs. fluoropyrimidine alone (group 3) regimens	Median DFS: 23.4, 14.1 and 16.3 mo, respectively (*p* = 0.088); HR group 1 vs. 3 = 0.63 (95% CI 0.39–1.03, *p* = 0.068); HR group 2 vs. 3 = 0.98 (95% CI 0.61–1.56, *p* = 0.918)	-	Median OS: 51.2, 47.9 and 60 mo, respectively (*p* = 0.219)
Portier et al., 2006 [48]	5-FU + folinic acid vs. SA	5-yr DFS: 33.5% vs. 26.7% (*p* = 0.028) OR = 0.66 (95% CI 0.46–0.96)	-	5-yr OS: 51.1% vs. 41.9% (*p* = 0.13); OR = 0.73 (95% CI: 0.48–1.10)
Mackay et al., 2005 [49]	Irinotecan	-	18-mo RFS 59% (95% CI: 43–80%)	2-yr OS 85% (95% CI, 72–99.8)
Gardini et al., 2004 [50]	TIL+IL-2 vs. SA	5-yr DSF: 21% vs. 31% (*p* = 0.27)	-	5-yr survival rate 25% vs. 38% (*p* = 0.7)

Abbreviations: 5-FU: 5-fluorouracil; 5-FU/LV: 5-fluorouracil/leucovorin; BEV: bevacizumab; CAPOX: capecitabine plus oxaliplatin; FOLFIRI: folinic acid, 5-FU plus irinotecan; FOLFOX: 5-FU/LV plus oxaliplatin; S-1: tegafur plus gimeracil and oteracil; TIL+IL2: tumor-infiltrating lymphocytes plus interleukin-2; UFT/LV: uracil/tegafur plus LV; AC: adjuvant chemotherapy; SA: surgery alone; DFS: disease-free survival; RFS: recurrence-free survival; OS: overall survival; NR: not reported; yr: year; mo: months.

**Table 4:** Outcomes of studies investigating adjuvant HAI chemotherapy.

Reference	Regimens of Chemotherapy	Outcomes		
		DFS	RFS	OS
Gholami et al., 2020 [51]	HAI (FUDR) vs. No HAI	-	5-yr RFS: 33% (28–38%) vs. 25% (20–30%) (*p* < 0.006); HR = 0.68 (95% CI 0.52–0.89); *p* < 0.005	5-yr OS 70% (65–75%) vs. 50% (43–57%), HR = 0.52 (*p* = 0.0001)
Buisman et al., 2020 [52]	HAI (FUDR) + systemic chemotherapy vs. systemic chemotherapy alone	median DFS: 20 months vs. 14 months, HR = 0.69 (95% CI 0.62–0.78, *p* < 0.001)	-	median OS: 84 vs. 57 months (HR 0.65, 95% CI 0.57–0.75, *p* < 0.001)
Goéré et al., 2013 [53]	HAI (oxaliplatin) + systemic 5-FU/LV vs. systemic irinotecan or oxaliplatin regimes alone	3-yr DFS 33% vs. 5% (*p* < 0.0001) HR = 0.37 (95% CI: 0.23–0.60)	-	3-yr OS 75% vs. 62% (*p* = 0.17); 5-yr OS 54% vs. 52% (*p* = 0.34)
House et al., 2011 [54]	HAI (FUDR) + systemic chemotherapy (5-FU/LV + irinotecan or oxaliplatin) vs. systemic chemotherapy alone	-	5-yr RFS: 48% vs. 25% (*p* < 0.01); HR = 0.71 (95% CI: 0.48–0.96)	5-yr DSS: 75% vs. 55% (*p* < 0.01); HR = 0.39 (95% CI: 0.23–0.68)
Alberts et al., 2010 [55]	HAI (FUDR) + systemic chemotherapy (oxaliplatin + capecitabine)	-	2-yr RFS 59.7% (48–74.3%)	2-yr OS 89.1% (81.2–97.7%)
Kemeny et al., 2002 [56]	HAI (FUDR) + systemic chemotherapy (5-FU)		4-yr RFS 45.7% vs. 25.2% (*p* = 0.4)	4-yr OS 61.5% vs. 52.7% (*p* = 0.6)
Tono et al., 2000 [57]	HAI (5-FU) vs. oral 5-FU	3-yr DFS: 66.7% vs. 20% (*p* = 0.045)		5-yr cumulative survival 77.8% vs. 50% (*p* = 0.2686)
Kemeny et al., 1999 [58]	HAI (FUDR) + systemic chemotherapy (5-FU + leucovorin) vs. systemic chemotherapy alone	-	-	2-yr OS 72% vs. 86%, *p* = 0.03; RR = 2.34 (1.10–4.98), *p* = 0.027
Rudroff et al., 1999 [59] ¹	HAI (mitomycin C/5-FU) vs. SA	Long-term DFS (group A vs. B): 23% vs. 15%	-	5-yr OS (group A vs. B): 31% vs. 25%
Lorenz et al., 1998 [60]	HAI (5-FU/FA) vs. SA	Relapse rate (18 m) 33.3% vs. 36.7% (*p* = 0.715)	-	median survival 34.5 m vs. 40.8 m (HR = 0.76, 95% CI 0.50–1.15, *p* = 0.1519)
Kokudo et al., 1998 [61]	Group 1: oral (UTF/5-FUDR) or IV (MMC or 5-FU) systemic chemotherapy vs. group 2: intra-arterial/intraportal regional therapy (5-FU/LV or MMC/5-FU/doxorubicin) vs. group 3: no adjuvant therapy	5-yr DFS: 33% vs. 26% vs. 19% (*p* = 0.02)	-	5-yr OS 51% vs. 49% vs. 37% (*p* = 0.37), RR (systemic vs. SA) = 0.202, RR (regional vs. SA) = 0.699 (*p* = 0.041)

Abbreviations: 5-FU: 5-fluorouracil; 5-FU/LV: 5-fluorouracil/leucovorin; UFT/LV: uracil/tegafur plus LV; FUDR: floxuridine; MMC: mitomycin C; SA: surgery alone; DFS: disease-free survival; RFS: recurrence-free survival; OS: overall survival; NR: not reported; yr: year. ^1^ The results from this study came from the comparison between groups A and B.

**Table 5:** Main severe toxicities in studies investigating adjuvant treatment after CRLM resection.

Reference	Severe Toxicities
Kanemitsu et al., 2021 [33]	AC: neutropenia: 50%, neuropathy: 10%, allergic reaction: 4%
Kokudo et al., 2021 [34]	Total: 12.2%, decreased Hb: 3.7%, diarrhea: 4.9%
Satake et al., 2021 [35]	Neutropenia: 29%
Nishioka et al., 2017 [38]	Oxaliplatin group: 50.9% UFT group: 6.8%
Kato et al., 2015 [39]	Neutropenia: 5%, fatigue: 6.7%
Kim et al., 2011 [44]	Neutropenia: 13.3%, anemia: 9.9%, thrombocytopenia: 11.6%, stomatitis: 8.3%, peripheral neuropathy: 3.3%
Alberts et al., 2010 [55]	HAI catheter/pump-related complications
Ychou et al., 2009 [46]	FOLFIRI: 47% (neutropenia 23%, diarrhea 14%), LV5-FU: 30% (neutropenia 7%, diarrhea 7%)
Portier et al., 2006 [48]	Diarrhea: 8.6%; hematologic, stomatitis, nausea: 7.4%; neuropathy: 2.47% (adjuvant chemotherapy arm)
Mackay et al., 2005 [49]	Neutropenia: 20.7%, diarrhea: 17.2%, vomiting: 13.8%
Gardini et al., 2004 [50]	Hyperpyrexia: 28,5%, oliguria: 14,2%
Kemeny et al., 2002 [56]	Increased liver enzymes: 30% (HAI arm)
Kemeny et al., 1999 [58]	Neutropenia: 21 vs. 18%, diarrhea: 14 vs. 29%, vomiting: 5 vs. 10%, bil > 3 mg/dL: 18% (HAI arm)
Lorenz et al., 1998 [60]	Stomatitis: 57.6 %, nausea: 55.4%, skin reaction: 26.9%, alopecia: 26.9%, pain: 24.9%, diarrhea: 23.6%
Kokudo et al., 1998 [61]	Unknown staging of complications

## Data Availability

Data available upon request from the corresponding author.

## Appendix A. Risk of Bias Assessment

**Table A1:** Risk of bias assessment of randomized clinical trials according to RoB2 tool.

Study	Randomization Process	Deviation from Intended Interventions	Missing Outcome Data	Measurement of Outcome	Selection of Reported Result	Overall
Kokudo et al., 2021 [34]	1.2 Y, 1.1 Y, 1.3 N LR	Part1: 2.1/2.2 Y, 2.3 PY, 2.4 PN SC Part2: 2.6Y LR	3.1 Y LR	4.1 N, 4.2 N, 4.3 Y, 4.4 PN LR	5.2/5.3 N 5.1 Y LR	SC
Kanemitsu et al., 2021 [33]	1.2 Y, 1.1 Y/PY, 1.3 N/PN LR	Part1: 2.1/2.2 Y, 2.3 PY, 2.4 PN SC Part2: 2.6Y LR	3.1 Y LR	4.1 N, 4.2 N, 4.3 Y, 4.4 PN LR	5.2/5.3 N 5.1 Y LR	SC
Kemeny et al., 2011 [43]	1.2 NI, 1.3 PN SC	Part1: 2.1/2.2 NI, 2.3 N LR Part2: 2.6 Y LR	3.1 Y LR	4.1 N, 4.2 N, 4.3 Y, 4.4 PN LR	5.2/5.3 N 5.1 Y LR	SC
Ychou et al., 2009 [46]	1.2 NI, 1.3 PN SC	Part1 : 2.1/2.2 Y, 2.3 PY, 2.4 PN SC Part2: 2.6N, 2.7 PN SC	3.1 Y LR	4.1 N, 4.2 N, 4.3 Y, 4.4 PN LR	5.2/5.3 N 5.1 Y LR	HR
Portier et al., 2006 [48]	1.2 NI, 1.3 PN SC	Part1: 2.1/2.2 Y, 2.3 PY, 2.4 PN SC Part2: 2.6Y LR	3.1 Y LR	4.1 PY HR	5.2/5.3 N 5.1 Y LR	HR
Kemeny et al., 2002 [56]	1.2 NI, 1.3 PN SC	Part1: 2.1/2.2 Y, 2.3 Y, 2.4 PN SC Part2: 2.6 N, 2.7 PY HR	3.1 Y LR	4.1 NI, 4.2 N, 4.3 Y, 4.4 PN LR	5.2/5.3 N 5.1 Y LR	HR
Tono et al., 2000 [57]	1.2 Y, 1.1 Y, 1.3 N LR	Part1 : 2.1/2.2 Y, 2.3 N LR Part2: 2.6 PY LR	3.1 Y LR	4.1 N, 4.2 N, 4.3 Y, 4.4 PN LR	5.2/5.3 N 5.1 Y LR	LR
Kemeny et al., 1999 [58]	1.2 NI, 1.3 PN SC	Part1: 2.1/2.2 Y, 2.3 Y, 2.4 NI, 2.5 NI HR Part2: 2.6 Y, 2.7 PY HR	3.1 Y LR	4.1 N, 4.2 N, 4.3 Y, 4.4 PN LR	5.2/5.3 N 5.1 Y LR	HR
Rudroff et al., 1998 [59] ¹	1.2 NI, 1.3 PN SC	Part1: 2.1/2.2 Y, 2.3 PN LR Part2: 2.6 PY LR	3.1 Y LR	4.1 NI, 4.2 Y HR	5.2/5.3 N 5.1 Y LR	HR
Lorenz et al., 1998 [60]	1.2 Y, 1.1 NI, 1.3 PN LR	Part1: 2.1/2.2 Y, 2.3 PY, 2.4 PN SC Part2 : 2.6Y LR	3.1 Y LR	4.1 N, 4.2 N, 4.3 Y, 4.4 PN LR	5.2/5.3 N 5.1 Y LR	SC

Abbreviations: LR: low risk; SC: some concern; HR: high risk. ^1^ The risk of bias assessment for this study focuses only on the comparison between groups A and B.

**Table A2:** Risk of bias assessment for non-randomized studies according to ROBINS-I tool.

Study	Confounding	Selection of Participants	Classifications of Interventions	Deviations from Intended Intervention	Missing Data	Measurement of Outcomes	Selection of Reported Result
Gardini et al. [50]	1.1 Y/1.2 N/1.4 N/1.6 N/1.7 N	2.1 Y/2.2 Y/2.3 PN	3.1 Y/3.2 Y/3.3 PN	4.1 Y/4.2 Y/4.3 NI/4.4 N/4.5 PY/4.6 N	5.1 Y/5.2 PN/5.3 PN	6.1 PN/6.2 PY/6.3 NI/6.4 PN	7.1 N/7.2 N/7.3 PN
	Risk of bias judgement: Serious	Risk of bias judgement: Serious	Risk of bias judgement: Low	Risk of bias judgement: Serious	Risk of bias judgement: Low	Risk of bias judgement: No information	Risk of bias judgement: Low

green: low risk; red: high risk.

**Table A3:** Risk of bias assessment according to Newcastle–Ottawa scale for cohort studies.

Study	Selection				Comparability		Outcome			Score
	Representativeness of the Exposure Cohort	Selection of the Non-Exposure Cohort	Ascertainment of Exposure	Outcome of Interest Not Present at the Start of the Study	Most Important Factor	Additional Factors	Assessment of Outcome	Follow-Up Was Long Enough	Adequacy of Follow-Up	
Sugimoto et al., 2021 [36]	1	1	1	1	1	1	1	1	0	8 (good)
Gholami et al., 2020 [51]	0	1	1	1	1	1	1	1	0	7 (good)
Buisman et al., 2020 [52]	1	1	1	1	1	1	1	1	1	9 (good)
Kobayashi et al., 2019 [37]	1	1	1	1	1	1	1	1	0	8 (good)
Nishioka et al., 2017 [38]	0	1	1	1	1	1	1	0	0	6 (poor)
Goéré et al., 2013 [53]	0	1	1	1	1	1	1	1	0	7 (good)
Hsu et al., 2013 [40]	0	1	1	1	1	1	1	1	1	8 (good)
Turan et al., 2013 [41]	1	1	1	1	1	1	1	1	1	9 (good)
House et al., 2011 [54]	0	1	1	1	1	1	1	1	0	7 (good)
Liu et al., 2010 [45]	0	1	1	1	1	1	1	1	1	8 (good)
Kim et al., 2009 [47]	1	1	1	1	1	1	1	1	1	9 (good)
Kokudo et al., 1998 [61]	0	1	1	1	1	1	1	0	0	6 (poor)

**Table A4:** Risk of bias assessment according to Newcastle–Ottawa scale for case–control studies.

Study	Selection				Outcomes		Exposure			Score
	Adequate Definition of Cases	Representativeness of the Cases	Selection of Controls	Definition of Controls	Most Important/Additional Factors		Ascertainment of Exposure	Same Method of Ascertainment	Non-Response Rate	
Kim et al., 2011 [44]	1	0	0	1	0	0	1	1	0	4 (poor)
